# Efficacy and safety of Telitacicept in IgA nephropathy and its impact on urinary Gd-IgA1: insights from a real-world study

**DOI:** 10.3389/fimmu.2026.1694197

**Published:** 2026-03-13

**Authors:** Hongfen Li, Wenying Li, Fanghao Wang, Yue Xing, Zhanfei Wu, Junya Jia, Youxia Liu, Tiekun Yan

**Affiliations:** 1 Department of Nephrology, Tianjin Medical University General Hospital, Tianjin, China; 2 Department of Nephrology, Tianjin Medical University General Hospital Airport Hospital, Tianjin, China

**Keywords:** efficacy, IgA nephropathy, safety, Telitacicept, urinary Gd-IgA1

## Abstract

**Objective:**

In this study, we evaluated the efficacy and safety of Telitacicept for treating patients with primary IgA nephropathy (IgAN) in our single hospital. We also explored the effect of Telitacicept on urinary Gd-IgA1 levels among patients with IgAN.

**Methods:**

A retrospective study was conducted. Patients were grouped according to 24-hour proteinuria (≥ 2.0 vs. < 2.0 g/day), eGFR (≥ 35 vs. < 35 mL/min/1.73 m^2^), gender (man vs. woman), age (≥ 35 vs. < 35 years) and therapy options (Telitacicept vs. Telitacicept plus glucocorticoid/immunosuppressor) to evaluate the therapeutic effect of Telitacicept on IgAN. We conducted a propensity score-matched comparison of the primary outcome between 20 IgAN patients with Telitacicept plus supportive treatment and 20 patients with supportive care alone. The urinary Gd-IgA1 levels were determined using ELISA.

**Results:**

A total of 68 IgAN patients who received Telitacicept were included in this study. At baseline, the median baseline proteinuria was 1753.5 mg/day, and eGFR was 71.5 ml/min/1.73m². Significant reductions in proteinuria were observed at 1 month and sustained through 6 months of follow-up. The eGFR remained stable throughout the follow-up period. Subgroup analyses stratified by baseline proteinuria, eGFR, gender, age, and therapy options showed no significant differences in proteinuria reduction rates. Both patients initially starting Telitacicept and those who had previously failed other therapy before starting it showed significant reductions in proteinuria and stable eGFR. Importantly, we observed an improvement in the eGFR slope within the Prior Treatment plus Telitacicept Group, with the annual rate of decline slowing from 6.35 ml/min/1.73m²/year pre-treatment to 3.68 ml/min/1.73m²/year after Telitacicept therapy. Furthermore, the responsive group to Telitacicept exhibited significantly higher IgA levels compared to the non-responsive group. Compared with patients receiving supportive care alone, those who initially added Telitacicept showed a greater reduction in proteinuria by the last follow-up. Additionally, Telitacicept therapy led to a decrease in urinary Gd-IgA1/Cr levels. Telitacicept treatment was well-tolerated.

**Conclusions:**

In IgAN, Telitacicept demonstrated promising efficacy, significantly reducing proteinuria and stabilizing eGFR, with a favorable safety profile.

## 1 Introduction

IgA nephropathy (IgAN) is indeed the most common primary glomerulonephritis globally, which primarily affects younger adults and can lead to significant kidney damage over time, resulting in a considerable socioeconomic burden due to the cost of treatment. Recent observational data from the UK indicates that even patients with modest proteinuria are at risk of developing kidney failure unless their annual rate of decline in kidney function is kept at or below 1 mL/min/1.73m^2^ (1, 2). Early and aggressive management of IgAN can help slow the progression of kidney damage and improve long-term outcomes for patients.

The current treatment options for IgAN lack standardization, with supportive measures such as sodium intake management, urine protein control, and blood pressure regulation forming the mainstay of therapy. In clinical practice, managing albuminuria and blood pressure levels typically involves utilizing angiotensin-converting enzyme inhibitors (ACEIs) or angiotensin receptor blockers (ARBs) (3). However, some patients are unable to tolerate these drugs due to their impaired kidney function or cough, which prevents them from using these medications or reaching the maximum dosage. Corticosteroids or a combination of immunosuppressants are prescribed for specific individuals. However, the potential adverse effects of steroids and immunosuppressive therapies may hinder their sustained long-term use (4). Current therapies fail to prevent kidney failure in a significant portion of patients, with incidence reaching 25% just 10 years after diagnosis. This concerning trend underscores the urgent need for innovative treatment approaches (5).

The accumulating body of evidence increasingly suggests that the pathogenesis of IgAN is intricately linked to autoimmunity (6). In recent years, numerous studies have demonstrated the pivotal role played by B cell survival factors such as B cell activating factors (BAFF), also known as B lymphocyte stimulators (BLyS), and a proliferation-inducing ligand (APRIL) in activating autoimmune signaling pathways. These factors have been shown to associate with the onset and severity of IgAN (7). Telitacicept is composed of two parts: the soluble fusion protein, which includes the transmembrane activator and calcium-modulating cyclophilin ligand interactor (TACI), along with the fragment crystallizable (Fc) domain of human IgG. The expected mechanism of action for Telitacicept involves inhibiting abnormal activation of B cells and plasma cells by antagonizing the interaction between BLyS or APRIL and their respective receptors on the surface of B lymphocytes. Targeting BAFF and APRIL emerges as a highly promising therapeutic approach for managing IgAN (4). However, to date, very limited studies have been conducted on the treatment of IgAN with Telitacicept.

In this study, we evaluated the efficacy and safety of Telitacicept for treating patients with primary IgAN in our single hospital. We also explored the effect of Telitacicept on urinary Gd-IgA1 levels among patients with IgAN.

## 2 Methods

### 2.1 Study design and population

**Figure 1:**
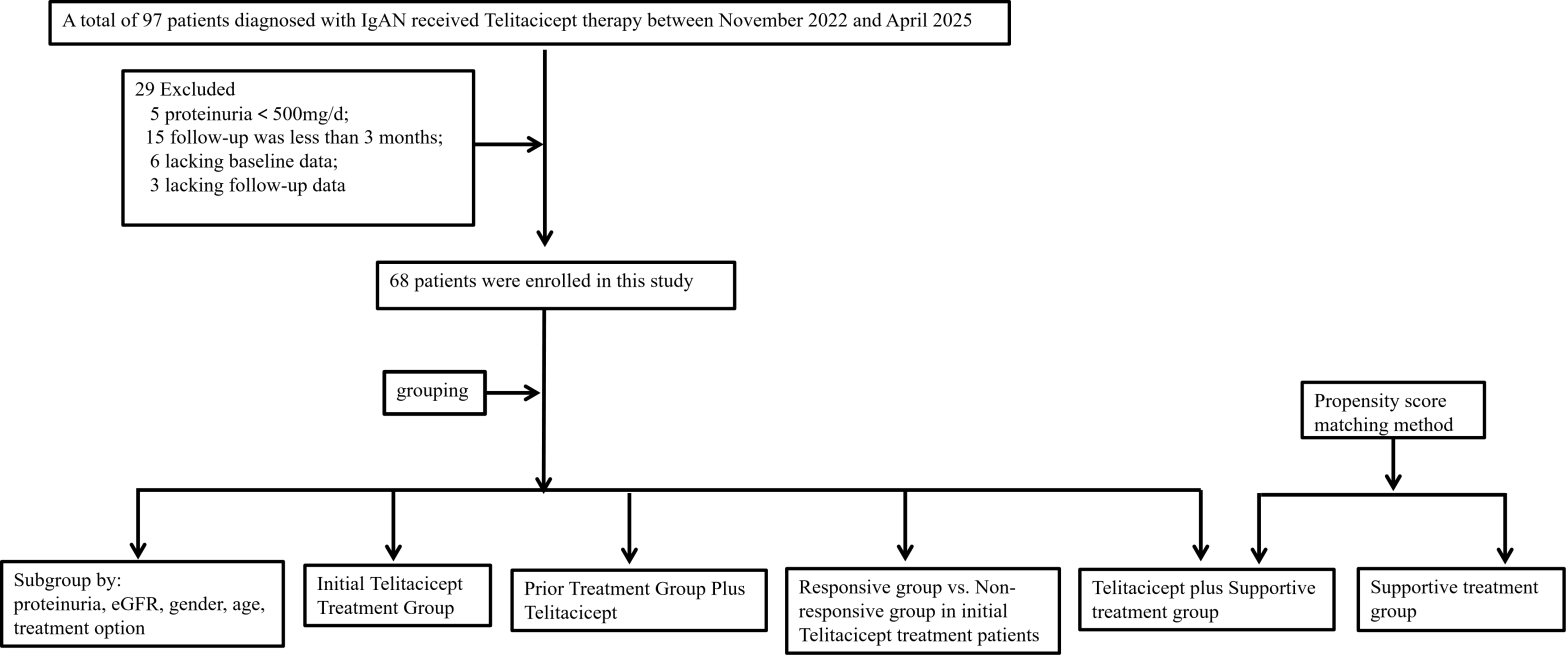
Flowchart of the recruitment process.

This retrospective study was conducted in a group of patients diagnosed with IgAN at Tianjin Medical University General Hospital. A total of 97 patients diagnosed with IgAN received subcutaneous Telitacicept 160 mg weekly between November 2022 and April 2025 and were enrolled in our study. Among these patients, a total of 29 IgAN patients were excluded. The exclusions included 5 patients with a 24-hour proteinuria level below 500 mg/day, 15 patients with an inadequate follow-up duration of less than 3 months, 6 patients lacking baseline data, and 3 patients lacking full follow-up data (**Figure 1**).

Of these participants, urine samples were obtained from 20 patients during the kidney biopsy phase and the follow-up phase.

We conducted a propensity score-matched comparison of the primary outcome between 20 patients with IgAN treated with supportive treatment alone and 20 patients treated with Telitacicept plus supportive treatment, all diagnosed during the same time period. We performed 1:1 propensity score matching on gender, age, blood pressure, renal function, and proteinuria to minimize baseline confounding and selection bias by balancing key prognostic factors and determinants of treatment response. This study was granted approval by the institutional ethics committee of Tianjin Medical University General Hospital (Ethics No. IRB2024-YX-216-01), and all patients provided written informed consent.

### 2.2 Clinical data collection

Demographic characteristics and clinical data, including age, gender, hypertension ratio, diabetic ratio, time from kidney biopsy to the initiation of Telitacicept treatment, follow-up duration after Telitacicept use, white blood cell count (WBC), hemoglobin level (Hb), neutrophil granulocyte count (NE), lymphocyte count, blood platelet count (PLT), serum albumin level (Alb), serum globulin level (GLO), serum creatinine level (Scr), estimated glomerular filtration rate (eGFR), serum uric acid level (UA), proteinuria, urinary red blood cell (URBC), total cholesterol level (TC), triglyceride level (TG), low-density lipoprotein level (LDL), serum immunoglobulin A concentration (IgA), serum immunoglobulin G concentration (IgG), Oxford classification (MEST-C scores) and drugs combination were collected at the time of initiation of Telitacicept treatment.

### 2.3 Outcomes

The primary outcome was defined as the change in proteinuria from baseline to follow-up. We set multiple secondary end points, including the cumulative frequencies of patients experiencing a 30% reduction in proteinuria, changes in eGFR, changes in hematuria, the proportion of proteinuria reducing to below 500 mg/day and below 300 mg/day and changes in urinary Gd-IgA1 levels.

### 2.4 Safety analysis

Safety data were collected through retrospective review of electronic medical records without a predefined systematic surveillance protocol. Adverse events were captured only when spontaneously reported by patients or documented by treating physicians during routine clinical care. Specific attention was given to injection site reactions, upper respiratory infections, and allergic reactions based on the known safety profile of Telitacicept.

### 2.5 Definitions

Individuals with a blood pressure of ≥ 140/90 mmHg are considered hypertension. The eGFR was determined using the Chronic Kidney Epidemiology Collaboration (CKD-EPI) equation, a widely utilized tool for assessing kidney function in patients with CKD (8). The histological lesions were categorized based on the Oxford classification scores (MEST-C, M: mesangial hypercellularity; E: endocapillary hypercellularity; S: segmental glomerulosclerosis; T: tubular atrophy/interstitial fibrosis, and C: crescent) (9). A renin-angiotensin system inhibitor (RASI) refers to the use of drugs such as ACEIs and/or ARBs after a biopsy. The rate of kidney function decline was expressed as the eGFR slope, which was calculated by fitting a straight line through eGFR values at all available time points using linear regression based on the least squares method (10). Supportive treatment (11) was defined as a maximum or tolerated dose of angiotensin-converting enzyme inhibitors (ACEIs), angiotensin receptor blockers (ARBs), sodium-glucose cotransporter 2 inhibitors (SGLT2is), and mineralocorticoid receptor antagonist (MRA). The non-responsive was defined as a reduction in proteinuria of less than 50% or end-stage renal disease (ESRD) achievement.

### 2.6 ELISA assay Gd-IgA1 levels

The Gd-IgA1 levels in urine were determined using ELISA according to the manufacturer’s protocol (KM55, IBL, German). Briefly, 100 μL of standard and diluents urine samples (1:2) was incubated for 1 hours at 37°C. After removing the liquid and washing 4 times, we added 100 μL labeled antibody for 1 h. Finally, the plates were incubated with TMB substrate for 15 mins, protected from light, followed by stopping the color development with the stop solution. The optical densities (ODs) were quantified at 450 nm with an EL32 Bio-Kinetics microplate reader (Bio-TekInstruments, Winooski, VT).

### 2.7 Statistical analysis

The ropensity score-matched comparison was conducted by 1:1 matching gender, age, blood pressure, renal function and proteinuria, with the matching caliper set at 0.2. Normally distributed continuous variables were compared using Student’s t test and expressed as mean ± standard deviation (SD). Non-normally distributed continuous data were compared using the Mann-Whitney U test and presented as medians and interquartile ranges. Dichotomous data were presented as both numerical values and percentages, and statistical comparison was performed using the χ2 test. Analyses were performed using the intention-to-treat (ITT) principle, with missing data handled by the last observation carried forward (LOCF) method. The statistical analysis, conducted using SPSS 25.0 software, indicated P < 0.05 (two-tailed) was considered significant.

## 3 Results

### 3.1 Baseline characteristics

A total of 68 IgAN patients who had accepted Telitacicept were included in this study. 10 patients discontinued treatment with Telitacicept. Among them, 3 patients initiated renal replacement therapy, 1 patient underwent kidney transplantation, 3 patients discontinued Telitacicept due to economic reasons, and 3 patients withdrew from treatment because of unsatisfactory efficacy. **Table 1** shows the participants’ baseline characteristics. There were 28 males among the patients. Additionally, 39 patients had hypertension. 5 patients had diabetes, but no histologic findings of diabetic nephropathy were observed when they underwent kidney biopsy. The mean age of the patients was (38.57 ± 13.28) years. The time from kidney biopsy to using Telitacicept was 0.50 (IQR, 0-28.75) months. The baseline laboratory values were as follows: WBC was (7.42 ± 2.35) ×10^9^/l, Hb was (131.38 ± 17.96) g/l, NE was (4.95 ± 1.97) × 10^9^/l, lymphocyte was (1.91 ± 0.63) × 10^9^/l, PLT was (257.26 ± 65.18) × 10^9^/l, ALB was (35.89 ± 5.59) g/l, Scr was 95.0 (IQR, 81.0 - 136.5) umol/l, and the eGFR was 71.51 (IQR, 47.13 - 88.55) mL/min/1.73m^2^. Baseline proteinuria was 1753 (IQR, 1171 - 3187) mg/day, and hematuria was 103.63 (IQR, 38.50 - 224.12)/uL. On the basis of the Oxford classification of IgAN, 64 (94%) patients had mesangial hypercellularity (M1), 44(65%) patients displayed endocapillary hypercellularity (E1), and 51(75%) patients showed segmental glomerulosclerosis (S1). For interstitial fibrosis/tubular atrophy (T) and crescent (C) lesions, T1 and T2 were found in 36 (53%) and 9 (13%) patients, respectively, whereas C1 and C2 were found in 38 (56%) and 11 (16%) patients, respectively.

**Table 1:** Baseline characteristics in IgAN patients with Telitacicept treatment (n = 68).

Variables	Values
Age (ys, mean ± SD)	38.57 ± 13.28
Gender (M%)	41.2
Hypertension, n (%)	39 (57.4)
Diabetics, n (%)	5 (7.3)
Time from renal biopsy to Telitacicept initiation (mon, median, IQR)	0.50 (0, 28.75)
Follow-up time after Telitacicept initiation (mon, median, IQR)	6 (6, 13)
WBC (×10*9/l, mean ± SD)	7.42 ± 2.35
Hb (g/l, mean ± SD)	131.38 ± 17.96
NE (×10*9/l, mean ± SD)	4.95 ± 1.97
Lymphocyte (×10*9/l, mean ± SD)	1.91 ± 0.63
PLT (×10*9, mean ± SD)	257.26 ± 65.18
ALB (g/l, mean ± SD)	35.89 ± 5.59
Scr (umol/l, median, IQR)	95.0 (81.0, 136.5)
eGFR (ml/min.1.73m^2^, median, IQR)	71.51 (47.13, 88.55)
UA (umol/l, mean ± SD)	398.71 ± 108.02
TC (mmol/l, mean ± SD)	5.07 ± 1.53
TG (mmol/l, mean ± SD)	2.03 ± 1.13
LDL (mmol/l, mean ± SD)	2.84 ± 1.08
Urine RBC (/ul, median, IQR)	103.63 (38.50, 224.12)
Proteinuria (mg/24h, median, IQR)	1753.5 (1171, 3187)
Total IgA (mg/dl, mean ± SD)	319.71 ± 114.97
Total IgG (mg/dl, mean ± SD)	936.32 ± 268.66
Pathological parameters, n (%)	
M (M0/M1)	4 (6) / 64 (94)
E (E0/E1)	24 (35) / 44 (65)
S (S0/S1)	17 (25) / 51(75)
T (T0/T1/T2)	23 (34) /36 (53) /9 (13)
C (C0/C1/C2)	19 (28) /38 (56) /11 (16)
Drug combination during Telitacicept treatment	
Prednisone or immunosuppressor, n (%)	35 (51.47)
RAASi, n (%)	49 (72.1)
SGLT-2 inhibitors, n (%)	33 (48.5)
MRAs, n (%)	5 (7.4)

M, man; F, female; SBP, systolic pressure; WBC, white blood cell; HB, hemoglobin; PLT, blood platelet; Scr, serum creatinine; eGFR, estimated glomerular filtration rate; Alb, albumin; TC, total cholesterol; TG, triglyceride; LDL, low density lipoprotein; C3, Complement 3; C4, Complement 4; Urine RBC, urinary red blood cell; M, mesangial hypercellularity; E, endocapillary hypercellularity; S, segmental glomerulosclerosis; T, tubular atrophy/interstitial fibrosis; C, crescent; RAASi, renin-angiotensin-aldosterone system inhibitor; SGLT2i, sodium-glucose cotransporter 2 inhibitor; MRA, mineralocorticoid receptor antagonist.

In terms of treatment modalities, the proportions of patients receiving concomitant medications were as follows: 35 (51.47%) patients were treated with a regimen that included prednisone and/or immunosuppressants; 49 (72.1%) patients underwent treatment with RAS blockers; 33 (48.5%) patients were administered a combination therapy that included SGLT-2 inhibitors; and 5 (7.4%) patients received a regimen that included MRAs. The follow-up time after using Telitacicept was 6 (IQR, 6-13) months (**Table 1**).

### 3.2 Primary outcomes

**Figure 2:**
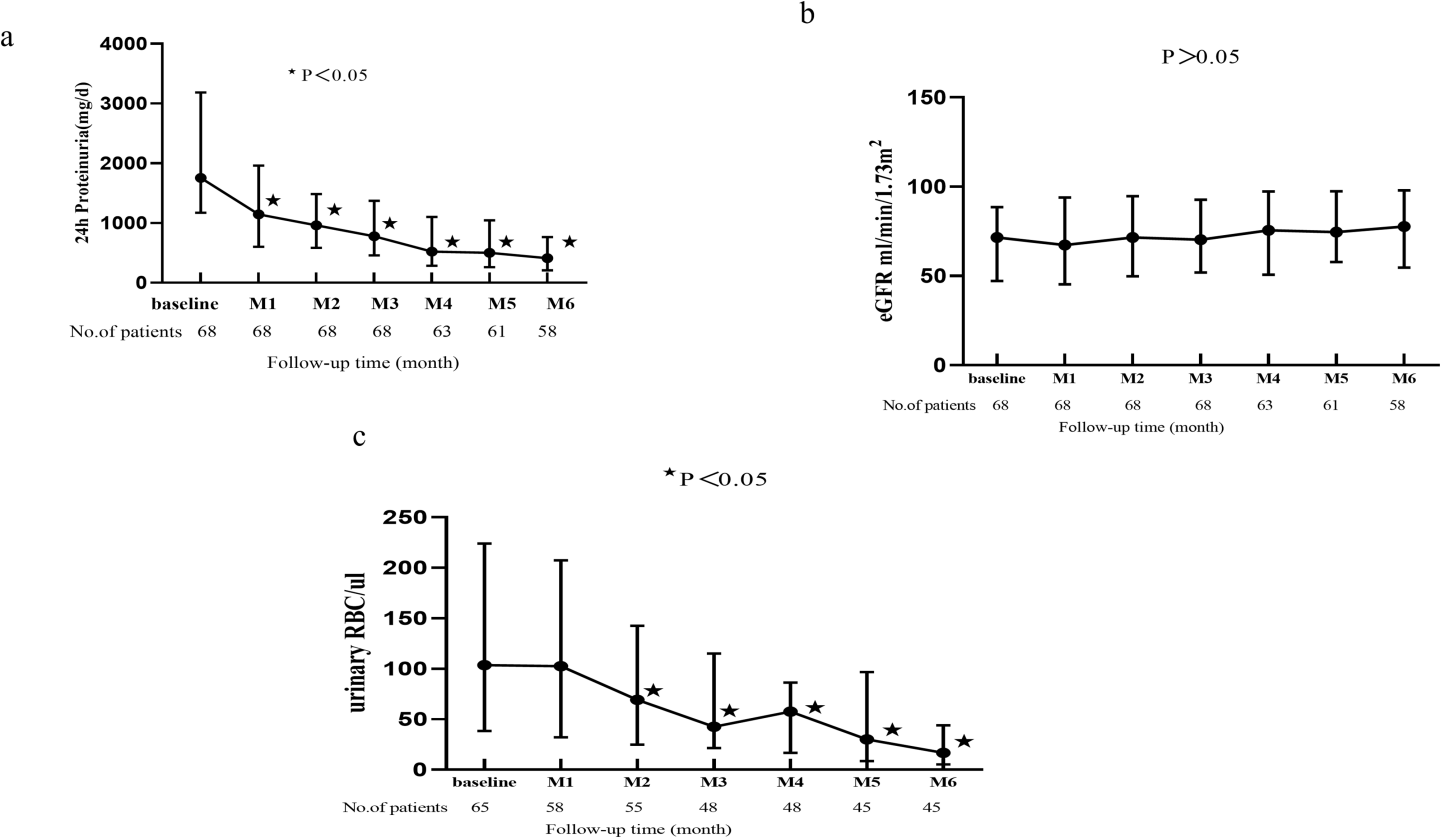
The change of **(A)** proteinuria, **(B)** eGFR and **(C)** hematuria during follow-up period in IgAN patients with Telitacicept treatment. Data are expressed as median, upper quartile and lower quartile. Comparisons between the follow-up time and the baseline were made every month. ★ means P<0.05.

A significant reduction in proteinuria was first observed at 1 month (1665 mg/day) and was sustained through 6 months of follow-up (370 mg/day; P < 0.001; **Figure 2A**). In the ITT population (n = 68), with missing 6-month urinary protein data imputed using the LOCF method, proteinuria was significantly reduced from a baseline median of 1753 mg/day to 423 mg/day at 6 months (P < 0.001; **Supplementary Figure 1**), consistent with the results in the per-protocol population (n = 58), supporting the robustness of the findings.

### 3.3 Secondary outcomes

A total of 63 (92.6%) patients demonstrated a proteinuria reduction rate exceeding 30%. Furthermore, 37 (54.4%) patients had their proteinuria reduced to below 500 mg/day, and 23 (33.8%) patients had their proteinuria reduced to below 300 mg/day (**Table 2**). The level of eGFR were comparable between at the baseline and 1–6 months of follow-up time (**Figure 2B** all P > 0.05). A significant reduction in hematuria was first observed at month 2 and sustained through month 6 of follow-up (**Figure 2C** all P < 0.05).

**Table 2:** Proteinuria reduction through the whole course in IgAN patients with Telitacicept treatment.

Variables	Values at last follow-up
Proteinuria reduction (mg, median, IQR)	1080 [574.3, 1978]
Proteinuria reduction rate (%, median, IQR)	69.27 [50.4, 83.7]
Proteinuria reduction rate ≥ 30%, n (%)	63 (92.6)
Proteinuria reducing to below 500 mg/day, n (%)	37 (54.4)
Proteinuria reducing to below 300 mg/day, n (%)	23 (33.8)

### 3.4 Subgroup analyses

#### 3.4.1 Primary outcomes by prespecified subgroup at baseline

**Figure 3:**
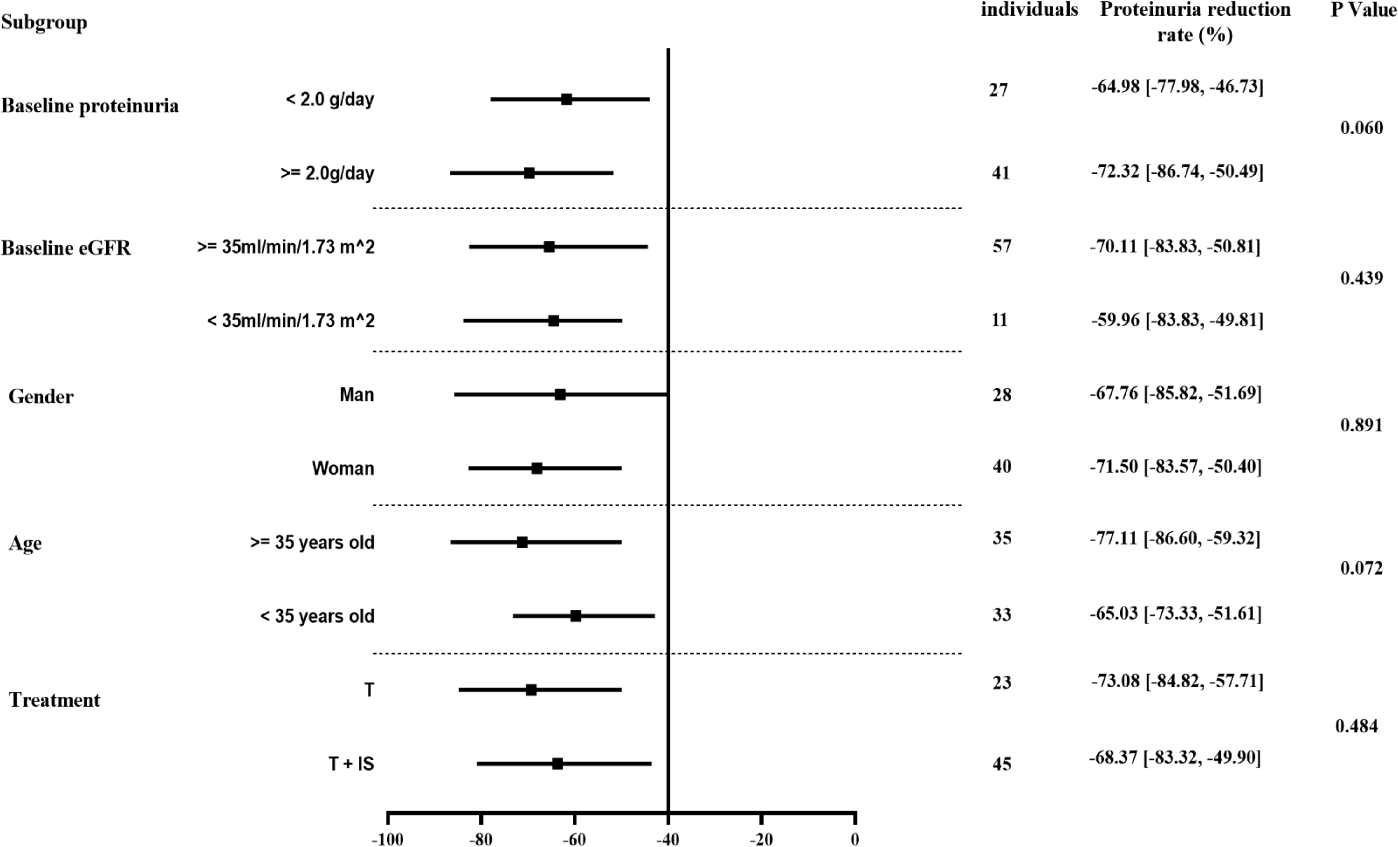
Comparison of urinary protein reduction rates among different subgroup in IgAN patients with Telitacicept treatment.

Subgroup analyses were based on different levels of baseline proteinuria (≥2.0 vs. <2.0 g/day), eGFR (≥35 vs. <35 mL/min/1.73 m^2^), gender (man vs. woman), age (≥35 vs. <35 years) and therapy options (T group: patients treated without glucocorticoid/immunosuppressor during Telitacicept treatment vs. T+IS group: patients treated with glucocorticoid/immunosuppressor during Telitacicept treatment). No significant differences in proteinuria reduction rates were observed between groups stratified by baseline proteinuria levels [64.98% (IQR, 46.73 - 77.98) vs. 77.32% (IQR, 50.49 - 86.74), P = 0.060], baseline eGFR levels [70.11% (IQR, 50.81 - 83.83) vs. 59.96% (IQR, 49.81-83.83), P = 0.439], gender [67.76% (IQR, 51.69 - 85.82) vs. 71.50% (IQR, 50.40 - 83.57), P = 0.891], or age [77.11% (IQR, 59.32 - 86.60) vs. 65.03% (IQR, 51.61 - 73.33), P = 0.072]. Similarly, no significant disparity was found between the T group and the T+IS group [73.08% (IQR, 57.71 - 84.82) vs. 68.37% (IQR, 49.90 - 83.32), P = 0.484], indicating consistent treatment efficacy regardless of these baseline characteristics or group assignments (**Figure 3**).

#### 3.4.2 The initial Telitacicept treatment group and the prior treatment plus Telitacicept group

**Figure 4:**
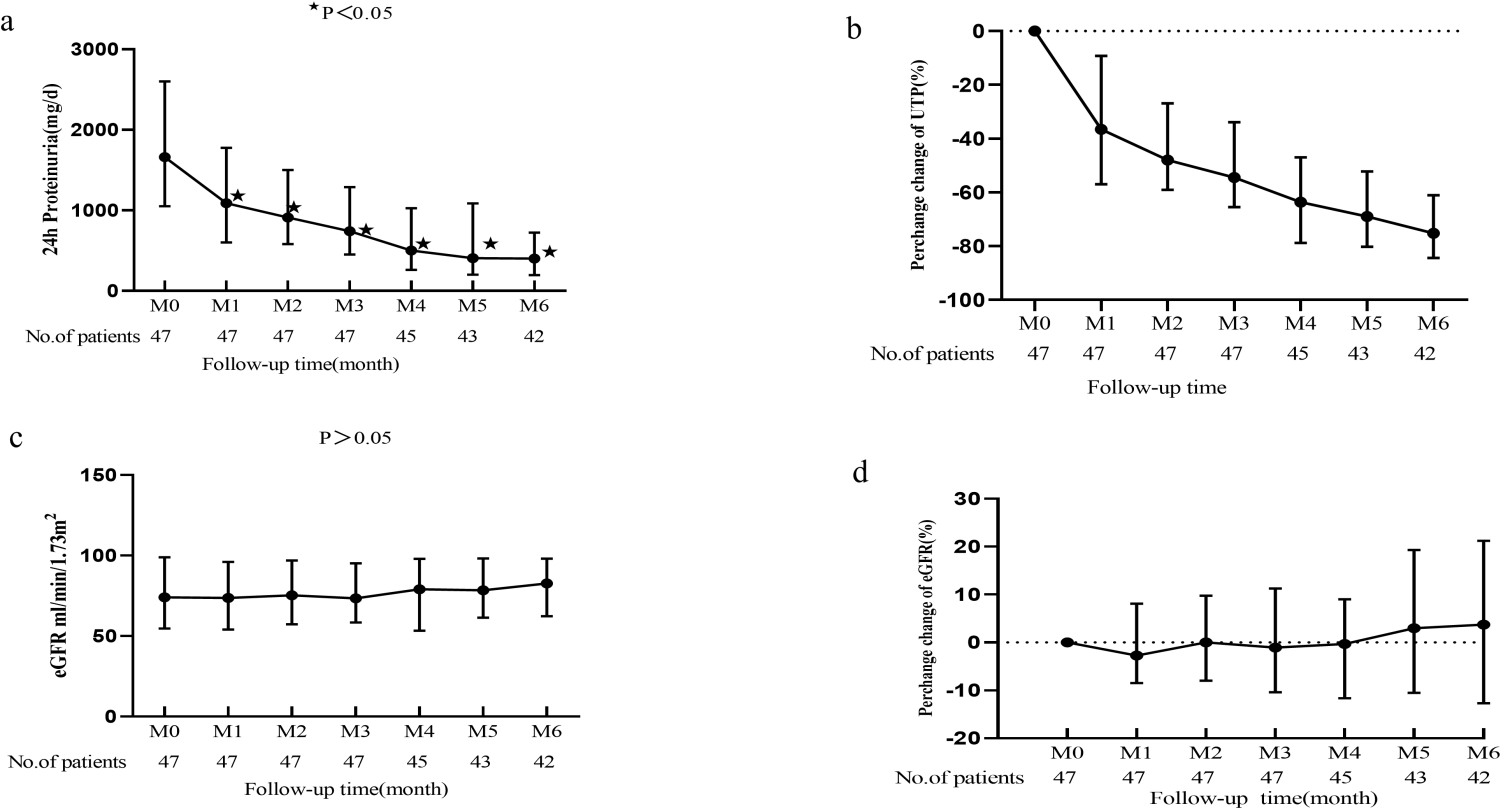
Trends in proteinuria and eGFR for IgAN patients in Initial Telitacicept Treatment Group. **(A)** Dynamic trends in proteinuria; **(B)** Median percentage changes in proteinuria; **(C)** Dynamic trends in eGFR; **(D)** Median percentage changes in eGFR from baseline (month 0, M0) to month 6 (M6). ★ means P<0.05.

**Figure 5:**
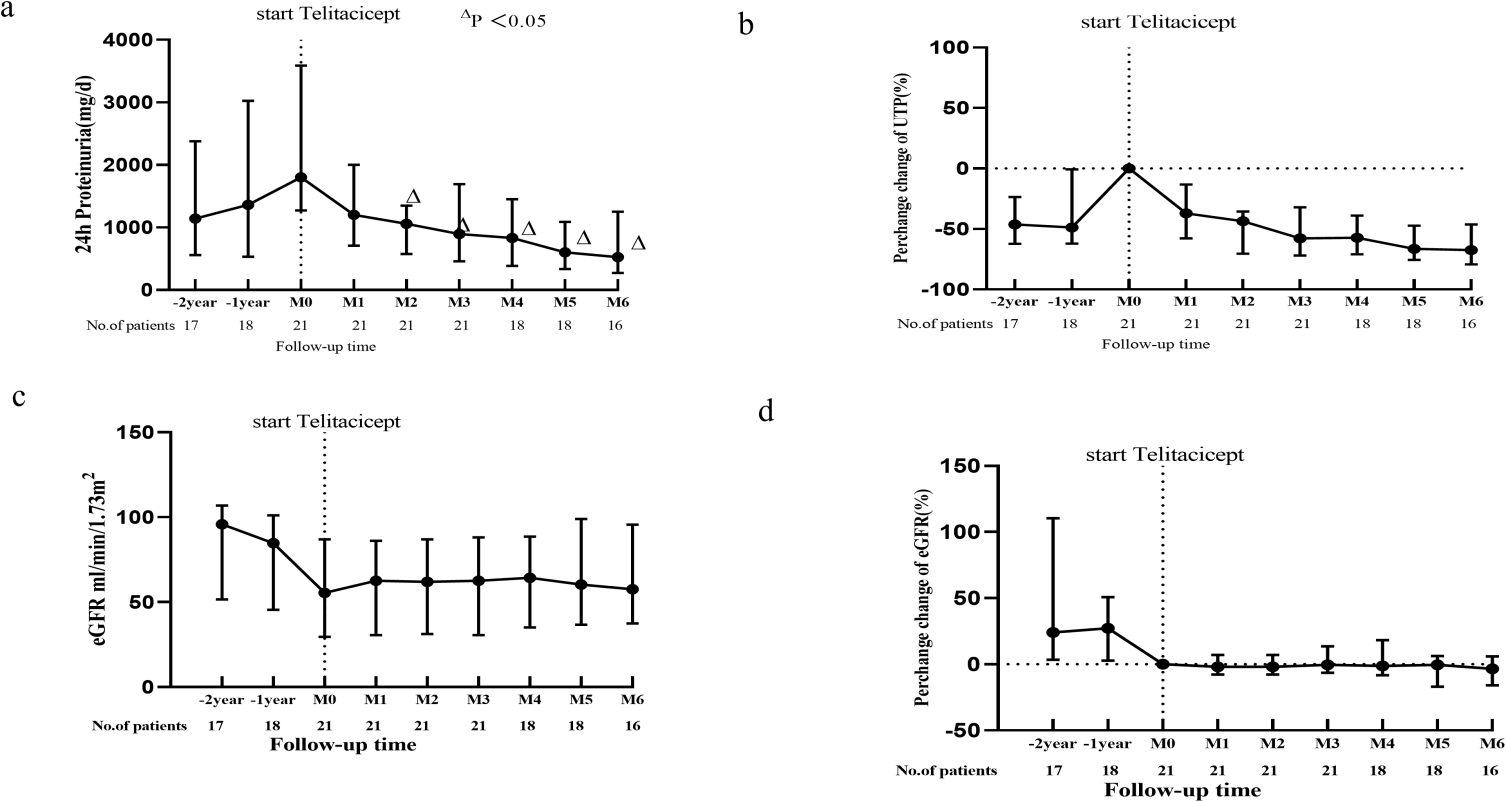
Trends in proteinuria and eGFR for IgAN patients in the Prior Treatment plus Telitacicept Group.**(A)** Dynamic trends in proteinuria; **(B)** Median percentage changes in proteinuria; **(C)** Dynamic rends in eGFR; **(D)** Median percentage changes in eGFR. Data are shown from 2 years prior to treatment initiation up to month 6 (M6), with all changes calculated relative to baseline (month 0, M0). △ means P<0.05.

Furthermore, the patients were stratified into two groups based on their treatment history following kidney biopsy: the Initial Telitacicept Treatment Group (n = 47), comprising patients who initiated Telitacicept therapy immediately after biopsy, and the Prior Treatment plus Telitacicept Group (n = 21), consisting of patients who had received other therapies beforehand. **Figures 4A, B** illustrate a significant reduction in proteinuria in the Initial Telitacicept Treatment Group by the first month of follow-up with this decrease sustained for 6 months. Furthermore, eGFR remained stable in the Telitacicept group throughout the treatment period (**Figures 4C, D**, P > 0.05). Within the Prior Treatment plus Telitacicept Group (n = 21), 18 patients had previously undergone glucocorticoid/immunosuppressive therapy, while the remaining 3 received only supportive care. Regarding follow-up duration, data were available for nearly 2 years for 17 patients and for nearly 1 year for 18 patients. As for the Prior Treatment plus Telitacicept Group, it showed a significant decrease in proteinuria, from 1800 (IQR, 1273 - 3587) to 1058 (IQR, 574 -1347) mg/day (P = 0.02) at the 2-month mark, an effect sustained for 6 months (**Figures 5A, B**). Importantly, we observed an improvement in the eGFR slope within the Prior Treatment plus Telitacicept Group, with the annual rate of decline slowing from 6.35 ml/min/1.73m²/year pre-treatment to 3.68 ml/min/1.73m²/year after Telitacicept therapy (**Figures 5C, D**).

#### 3.4.3 Responsive group vs. non-responsive group in initial Telitacicept treatment patients

Among the 47 patients with initial treatment, 9 had a proteinuria reduction of no more than 50% from baseline at the last follow-up, and 2 progressed to ESRD. These 11 patients were categorized as the non-responsive group, while the remaining 36 patients were categorized as the responsive group. As shown in **Table 3**, there were no significant differences between the two groups in terms of gender, age, prevalence of hypertension and diabetes, baseline proteinuria, eGFR, ALB, Urine RBC, Total IgG, C3, C4 TC, TG, LDL or Oxford classification of MESTC lesions (all P > 0.05). Interesting, the responsive group exhibited significantly higher IgA levels than the non-responsive group (342.72 ± 112.86 vs. 249.38 ± 87.37 mg/dl, P = 0.049).

**Table 3:** Comparison of baseline characteristics between Responsive and Non-responsive group in IgAN patients with initial Telitacicept treatment.

Variables	Non-responsive group (n = 11)	Responsive group (n = 36)	P value
Gender (M/F)	5/6	14/22	0.698
Age (ys, mean ± SD)	36.18 ± 9.89	38.50 ± 14.62	0.626
Hypertension, n (%)	8 (72)	18 (50)	0.185
Diabetics, n (%)	1 (9)	3 (8)	0.937
Baseline proteinuria (mg/d, median, IQR)	1451[500, 5824]	1699 [1098, 2586]	0.951
Baseline eGFR (ml/min/1.73m^2^, median, IQR)	88.03 [37.65, 102.51]	71.88 [54.70, 87.89]	0.545
ALB (g/l, mean ± SD)	34.00 ± 7.54	35.89 ± 4.61	0.327
TC (mmol/l, mean ± SD)	5.80 ± 2.08	4.94 ± 1.45	0.159
TG (mmol/l, mean ± SD)	2.21 ± 0.98	1.85 ± 0.83	0.261
LDL (mmol/l, mean ± SD)	3.37 ± 1.42	2.76 ± 1.00	0.146
Urine RBC (/ul, median, IQR)	126.50 [14.30, 298.60]	96.20 [41.20, 242.60]	0.859
Total IgA (mg/dl, mean ± SD)	249.38 ± 87.37	342.72 ± 112.86	0.049
Total IgG (mg/dl, mean ± SD)	808.50 ± 319.66	958.21 ± 229.15	0.237
C3 (mg/dl), mean ± SD)	96.65 ± 19.55	92.53 ± 19.86	0.652
C4 (mg/dl, mean ± SD)	24.07 ± 4.69	27.21 ± 7.91	0.366
Pathological parameters, n (%)			
M1	9 (81.8)	34 (94.4)	0.189
E1	7 (63.6)	22 (61.1)	0.880
S1	8 (72.7)	26 (72.2)	0.974
T1+T2	8 (72.7)	22 (61.1)	0.483
C1+C2	7 (63.6)	25 (69.4)	0.718

M, man; F, female; eGFR, estimated glomerular filtration rate; Alb, albumin; TC, total cholesterol; TG, triglyceride; LDL, low density lipoprotein; Urine RBC, urinary red blood cell; M, mesangial hypercellularity; E, endocapillary hypercellularity; S, segmental glomerulosclerosis; T, tubular atrophy/interstitial fibrosis; C, crescent.

#### 3.4.4 Telitacicept group vs. supportive treatment group

Additionally, we conducted a propensity score-matched comparison of the primary outcome between 20 patients with IgAN treated with Telitacicept plus supportive treatment and 20 patients treated with supportive care alone based on age, sex, baseline proteinuria, eGFR level and follow-up time. The results presented in **Table 4** provide insights into the outcomes at the end of the final follow-up period. In the Telitacicept plus Supportive Treatment Group, a significant difference in proteinuria reduction rate was found between the two groups [75.29% (58.12, 81.83) vs. 47.55% (4.33, 68.44), P = 0.003]. The eGFR was comparable between the two groups [97.41(78.44, 107.39) vs. 81.58(71.50, 96.60) ml/min/1.73m^2^, P = 0.090].

**Table 4:** Comparison of characteristics between patients with Telitacicept plus Supportive treatment and Supportive treatment alone after matching in patients with IgAN.

Variables	Telitacicept plus Supportive treatment (n = 20)	Supportive treatment (n = 20)	P value
Gender (M/F)	3/17	5/15	0.429
Age (ys, mean ± SD)	35.35 ± 11.49	39.60 ± 8.19	0.186
Hypertension, n (%)	8 (40)	7 (35)	0.744
Diabetics, n (%)	3 (15)	4 (20)	0.677
Baseline proteinuria (mg/d, median, IQR)	1437 [853, 1719]	1087 [859, 1341]	0.337
Baseline eGFR (ml/min/1.73m^2^, median, IQR)	81.49 [68.30, 111.04]	83.77 [72.29, 100.05]	0.758
ALB (g/l, mean ± SD)	37.30 ± 4.03	37.45 ± 3.95	0.905
Pathological parameters, n (%)			
M1	19 (95.0)	16 (80.0)	0.151
E1	10 (50.0)	9 (45.0)	0.752
S1	17 (85.0)	16 (80.0)	0.677
T1+T2	12 (60.0)	11 (55.0)	0.943
C1+C2	10 (50.0)	10 (50.0)	0.875
Final follow-up characteristics			
Follow-up time (mon, median, IQR)	11.0 [6.0, 13.0]	9.5 [6.0, 13.0]	0.912
eGFR (ml/min/1.73m2, median, IQR)	97.41[78.44, 107.39]	81.58[71.50, 96.60]	0.090
Proteinuria (mg/d, median, IQR)	287 [163, 611]	616 [400, 900]	0.009
Proteinuria reduction rate (%, median, IQR)	75.29 [58.12, 81.83]	47.55 [4.33, 68.44]	0.003

M, man; F, female; eGFR, estimated glomerular filtration rate; Alb, albumin; M, mesangial hypercellularity; E, endocapillary hypercellularity; S, segmental glomerulosclerosis; T, tubular atrophy/interstitial fibrosis; C, crescent.

#### 3.4.5 Percentage change of the urinary Gd-IgA1 after Telitacicept treatment

**Figure 6:**
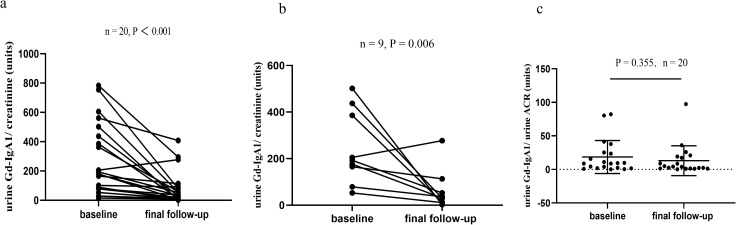
Urinary Gd-IgA1 Trends in Telitacicept treated IgAN patients. **(A)** Changes in urinary Gd-IgA1/creatinine (mg/g) at baseline and final follow-up in 20 Telitacicept-treated IgAN patients; **(B)** Changes in urinary Gd-IgA1/creatinine (mg/g) at baseline and final follow-up in 9 remaining patients (after exclusion of those receiving glucocorticoids/immunosuppressants); **(C)** Changes in urinary Gd-IgA1/ACR (mg/g) at baseline and final follow-up in 20 Telitacicept-treated IgAN patients.

As shown in **Figures 6A, B**, Telitacicept treatment led to a decrease in urinary Gd-IgA1/Cr levels. At the last follow-up, these levels decreased from 186.30 (IQR, 81.22 - 485.64) to 33.73 (IQR,10.99 - 87.90) mg/g (**Figure 6A**, P < 0.001). Even after excluding patients treated with glucocorticoids/immunosuppressive agents, Telitacicept still lowered urinary Gd-IgA1/Cr levels from 193.73 (IQR, 122.09 - 411.52) to 30.91 (IQR,11.99 - 83.05) mg/g (**Figure 6B**, P = 0.006). Urinary Gd-IgA1 normalized to ACR trended downward after treatment, but this change did not reach statistical significance (**Figure 6C**, P = 0.355).

### 3.5 Side effects

Telitacicept was tolerated by most patients. No severe AE was reported. Ten patients (14.7%) reported AEs, including injection site reactions (N = 8), upper respiratory infections (N = 2), no patients experienced injection-site allergic-related rash. No one withdrew Telitacicept because of AEs.

## 4 Discussion

In this real-world retrospective study, the findings demonstrated the potential efficacy of Telitacicept in reducing proteinuria and hematuria in patients with IgAN while maintaining a stable eGFR. Furthermore, the observed injection site reactions are noteworthy, although it’s positive that there wasn’t an increase in the risk of serious infections among patients treated with Telitacicept. This information contributes to the safety profile of Telitacicept, which is a promising therapeutic option for managing IgAN.

Recent findings from a phase II clinical trial (NCT04291781) (12) have shown that continuous administration of Telitacicept at a dosage of 240 mg per week for 24 weeks results in a significant reduction in average 24-hour proteinuria levels by 49% compared to baseline. In contrast, a dosage of 160 mg per week reduced proteinuria by 25%, which was not statistically significant. Additionally, patients receiving Telitacicept at either 160 mg or 240 mg per week demonstrated a statistically significant increase in the eGFR compared to the placebo group, indicating an improvement in kidney function. In present study, we found there was a significant reduction in total urine protein levels in Telitacicept 160 mg per week treatment, which was also significant. The baseline proteinuria was generally comparable between studies (median 1753 mg/day vs. median 1630 mg/day in the 160 mg group of Lv et al.), and renal function (median eGFR 71.5 vs. 71.67 mL/min/1.73m²) was comparable. The apparent discrepancy in proteinuria reduction between our study and the phase II trial by Lv et al (12). can be explained by differences in treatment protocols. First, concomitant supportive therapy differed substantially: 72.1% of our patients received RAS blockers and 48.5% received SGLT2 inhibitors, whereas the use of SGLT2 inhibitors was not clearly described in Lv’s trial. Second, 51.47% of our patients received Telitacicept in combination with prednisone and/or immunosuppressants, whereas Lv’s trial included patients with prior immunosuppressant exposure but did not allow concurrent use during the study period. The more intensive background supportive care and combination immunosuppressant therapy in our real-world cohort likely contributed to the more pronounced proteinuria reduction observed with the 160 mg dose in our study compared with the 160 mg dose group in Lv et al. Currently, the initiation of treatment for patients with IgAN is determined by proteinuria levels and eGFR, which are late indicators of kidney damage. To assess the therapeutic efficacy of Telitacicept in IgAN under different conditions, patients were categorized based on their baseline eGFR, 24-hour proteinuria levels, gender, age, and treatment options. Our subgroup analysis revealed no significant difference in the decline in proteinuria across different levels of eGFR at baseline. Our previous study (13) suggested that patients with severe kidney insufficiency (stage 4 and 5 CKD) experienced a poorer prognosis under current therapies. This underscores the urgent need for new drugs and therapeutic strategies to improve outcomes for these patients with advanced CKD. It seems that Telitacicept is regarded as an alternative or rescue therapy in clinical practice even for late-stage IgAN by physicians. Although Telitacicept shows promise in reducing proteinuria in patients with severe kidney insufficiency, further evidence is needed to establish its efficacy and potential benefits in this population. Our analysis indicates that urinary protein levels decreased by 72.32% and 64.98% in patients with baseline proteinuria of ≥ 2.0 g/d and < 2.0 g/d, respectively. This indicates Telitacicept’s potential effectiveness in reducing proteinuria regardless of baseline level. Interestingly, proteinuria reduction was comparable between the T group and the T+IS group. Patients in the T+IS group had more severe and active disease at baseline, which justified the addition of immunosuppressive therapy; notably, 40% of these patients were switched to this combined regimen due to an inadequate response to prior immunosuppression. The finding that this higher−risk population achieved a similar degree of proteinuria reduction to the T group suggests that Telitacicept remains effective even in more difficult−to−treat patients. Thus, the absence of a significant between−group difference represents a favorable efficacy signal, supporting that Telitacicept is effective both as first−line therapy and as a valuable rescue treatment for refractory IgAN. In phase II trials of Telitacicept for IgAN with persistent proteinuria (12), 4 patients in the 160 mg group had a history of systemic corticosteroid therapy, while 4 patients had a history of other immunosuppressant therapy. In the 240 mg group, there were 5 patients with a history of other immunosuppressant therapy. Following the administration of Telitacicept, both groups experienced reduction in proteinuria by 25% and 49%, respectively. Similarly, the present results (14, 15) suggest that Telitacicept shows promise for reducing proteinuria in IgAN patients, both as an initial treatment and for those previously treated with corticosteroids or immunosuppressants after kidney biopsy.

We compared treatment responses in patients initially treated with Telitacicept. Interestingly, the response group exhibited higher IgA levels, suggesting that robust B cell activity leading to increased IgA production might represent a condition conducive to Telitacicept’s mechanism of action. Furthermore, our results showed that blood lipid levels were higher in the non-responsive group compared to the responsive group, indicating that patients presenting with early-stage hyperlipidemia often face greater treatment challenges. It’s also important to control lipid levels in IgAN patients.

Currently, several supportive treatments for IgAN, including RAAS inhibitors, SGLT-2 inhibitors, and MRAs, can be chosen to manage proteinuria. Often, these supportive measures alone are sufficient to achieve proteinuria remission. However, this raises the question: why might we consider initiating Telitacicept therapy? In our matched study, we compared 20 patients receiving Telitacicept plus supportive treatment with 20 IgAN patients diagnosed during the same period and treated with supportive treatment alone. We found a significant difference in the proteinuria reduction rate between the two groups over 6 months: the Telitacicept group showed a median reduction of 75.29% (58.12, 81.83) compared to 46.38% (2.77, 64.48) in the Supportive Treatment Group (P = 0.001). Additionally, the eGFR in the Telitacicept group was slightly higher than that in the Supportive Treatment Group, although the difference did not reach statistical significance [97.41 (78.44, 107.39) vs. 83.79(71.63, 101.26) ml/min/1.73m^2^, P = 0.205]. These results suggest that supportive treatment combined with Telitacicept may lead to faster proteinuria reduction and more stable GFR, potentially offering better therapeutic outcomes for IgAN patients. However, more patients and longer follow-up are needed to confirm these initial findings and determine its optimal clinical application.

A previous study by Lv et al (16). demonstrated that Telitacicept lowers circulating Gd-IgA1 levels. Building on this, our study further explored Telitacicept’s effect on urinary Gd-IgA1 levels. We found that Telitacicept significantly reduced urinary Gd-IgA1/Cr ratios in patients with IgAN. Although glucocorticoids or immunosuppressants may also lower Gd-IgA1 levels, a sensitivity analysis excluding patients receiving these agents confirmed that Telitacicept remained effective in reducing urinary Gd-IgA1/Cr. Studies from Japanese and Korean cohorts have demonstrated that urinary excretion of Gd-IgA1-when normalized to creatinine (Gd-IgA1/Cr) or protein-to-creatinine ratio (Gd-IgA1/PCR)-is significantly elevated in patients with IgAN relative to disease controls and healthy individuals (17, 18). Although urinary Gd-IgA1 normalized to ACR only trended downward without statistical significance, Telitacicept treatment nonetheless resulted in a 73% reduction in proteinuria, compared with an 82% reduction in urinary Gd-IgA1/Cr in these 20 IgAN patients. This disproportionate reduction in Gd-IgA1 excretion raises the possibility of a treatment effect specific to Gd-IgA1 production or clearance, though we cannot exclude confounding by overall proteinuria reduction or establish causality from this observational study. Future larger studies will be required to formally confirm whether this represents a specific drug effect on Gd-IgA1. Future larger studies will be required to formally confirm causality and further clarify whether changes in urinary Gd-IgA1 may serve as an early predictive biomarker of therapeutic response in IgAN.

According to previous studies (19–23), adverse events associated with Telitacicept predominantly including injection site reactions, upper respiratory infections, and allergies. By following these patients for 6 months, no severe infections or serious adverse events leading to discontinuation were observed. No severe AE was reported. Only ten patients reported AEs, including injection site reactions and upper respiratory infections. No one withdrew Telitacicept because of AEs. This suggested a favorable safety profile for Telitacicept in this patient population.

Several limitations are present in this study. First, this was a retrospective real-world study, which inherently carries the risk of selection bias. Second, the relatively small sample size and short follow-up period may limit the statistical power of our analyses and the generalizability of our findings to the broader IgAN population. Third, the majority of patients received Telitacicept in combination with other immunosuppressive agents. Therefore, we cannot fully disentangle the independent therapeutic contribution of Telitacicept from the effects of concomitant immunosuppressive therapies. Furthermore, the safety analysis was limited by the retrospective design without systematic adverse event monitoring. Future prospective studies with standardized safety assessments are needed to fully characterize the safety profile of Telitacicept.

## 5 Conclusion

Telitacicept demonstrated promising efficacy in reducing proteinuria and stabilizing GFR in IgAN. Furthermore, it exhibited a lower incidence of adverse effects, highlighting its potential as a therapeutic option for this condition. However, these findings require validation through larger-scale studies with longer follow-up in the future.

## Data Availability

The raw data supporting the conclusions of this article will be made available by the authors, without undue reservation.

## Supplementary material

Supplementary Figure 1 Changes in proteinuria at baseline and final follow-up in IgAN patients with Telitacicept treatment according to intention-to-treat analysis.
