# Supplementary figures and images for: Efficacy and safety of Telitacicept in IgA nephropathy and its impact on urinary Gd-IgA1: insights from a real-world study
Source: Front Immunol. 2026 Mar 13;17:1694197. doi: 10.3389/fimmu.2026.1694197 (PMC13022593; doi:10.3389/fimmu.2026.1694197)

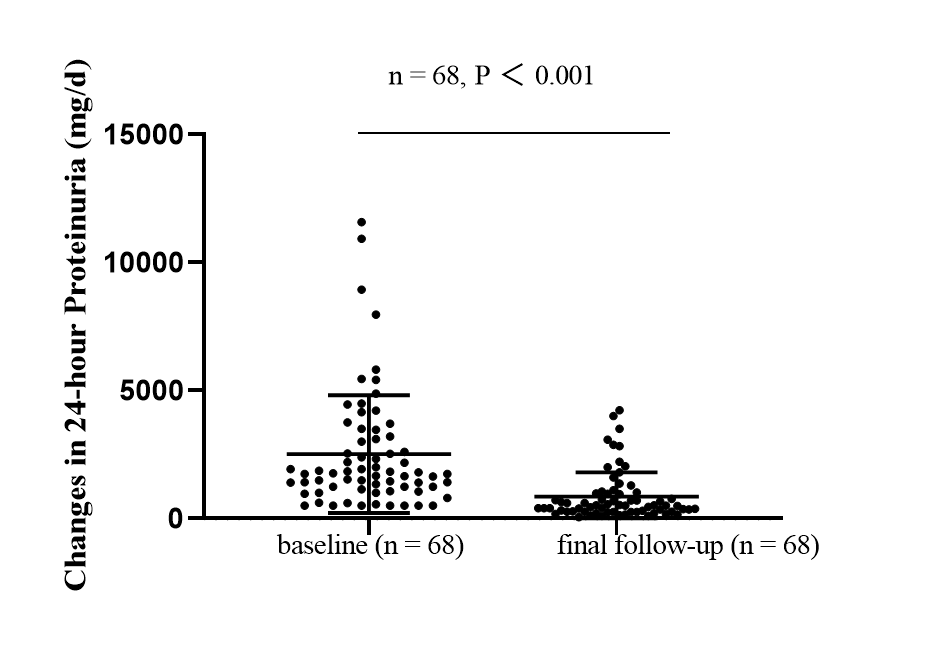

Supplement: Supplementary Figure 1 — Changes in proteinuria at baseline and final follow-up in IgAN patients with Telitacicept treatment according to intention-to-treat analysis. [file Image1.tif]
